# Inhibition of N-Type Calcium Channels by Fluorophenoxyanilide Derivatives

**DOI:** 10.3390/md13042030

**Published:** 2015-04-13

**Authors:** Ellen C. Gleeson, Janease E. Graham, Sandro Spiller, Irina Vetter, Richard J. Lewis, Peter J. Duggan, Kellie L. Tuck

**Affiliations:** 1 School of Chemistry, Monash University, Clayton VIC 3800, Australia; E-Mails: ellen.gleeson@monash.edu (E.C.G.); janease.graham@gmail.com (J.E.G.); sandro.spiller@bbz.uni-leipzig.de (S.S.); 2 CSIRO Manufacturing Flagship, Clayton South, VIC 3169, Australia; 3 Institute for Molecular Bioscience, The University of Queensland, St Lucia QLD 4072, Australia; E-Mails: i.vetter@imb.uq.edu.au (I.V.); r.lewis@imb.uq.edu.au (R.J.L.); 4 School of Chemical and Physical Sciences, Flinders University, Adelaide SA 5042, Australia

**Keywords:** N-type calcium channel, Ca_v_2.2, channel blocker, pain, FLIPR

## Abstract

A set of fluorophenoxyanilides, designed to be simplified analogues of previously reported ω-conotoxin GVIA mimetics, were prepared and tested for N-type calcium channel inhibition in a SH-SY5Y neuroblastoma FLIPR assay. N-type or Ca_v_2.2 channel is a validated target for the treatment of refractory chronic pain. Despite being significantly less complex than the originally designed mimetics, up to a seven-fold improvement in activity was observed.

## 1. Introduction

Neuropathic pain, which results from nerve damage caused by surgery, trauma, infection or disease, often does not respond to existing therapies [1,2]. Various estimates put the proportion of the world’s population afflicted by this condition to be at least 3%, with up to 5% of postoperative patients being affected. Safe and effective therapies for neuropathic pain are therefore a major unmet medical need. N-Type calcium channels (Ca_v_2.2 channels) are strongly implicated in chronic and neuropathic pain and their inhibitors have been widely pursued [1,2,3,4]. This approach has been best validated by Ziconotide (Prialt^®^), a synthetic version of the peptide ω-conotoxin MVIIA found in the venom of a fish-hunting marine cone snail *Conus magnus*. This peptide selectively targets Ca_v_2.2 channels and is one of the very few effective drugs used to treat intractable chronic pain [5]. However, its intrathecal mode of delivery and narrow therapeutic window make it less than ideal as a treatment option.

**Figure 1:**
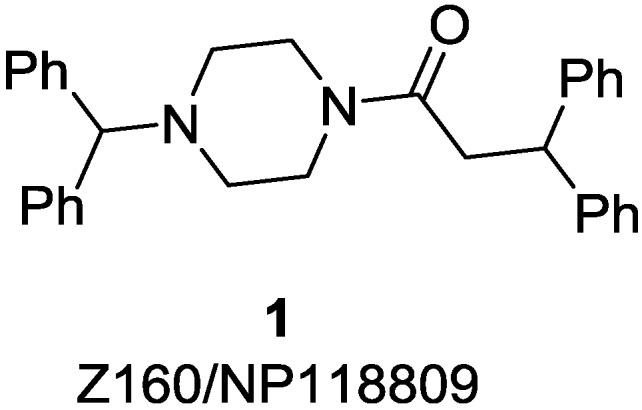
Chemical structure of Z160/NP11809 (**1**).

We as well as others have been developing small-molecule inhibitors of Ca_v_2.2 channels as possible alternatives to Ziconotide [6,7,8,9,10,11,12,13,14,15,16,17,18,19,20,21,22,23,24,25,26,27,28,29]. Recently clinical development of Z160 (**1**, Figure 1), a reformulated form of NP118809 [8], was discontinued after Z160 (**1**) failed to meet the primary endpoint in Phase II clinical studies [30]. As a result there is only one compound that targets Ca_v_2.2 channels currently in clinical trials for the treatment of chronic pain, CNV2197944, the structure of which is yet to be disclosed [24].

**Figure 2:**
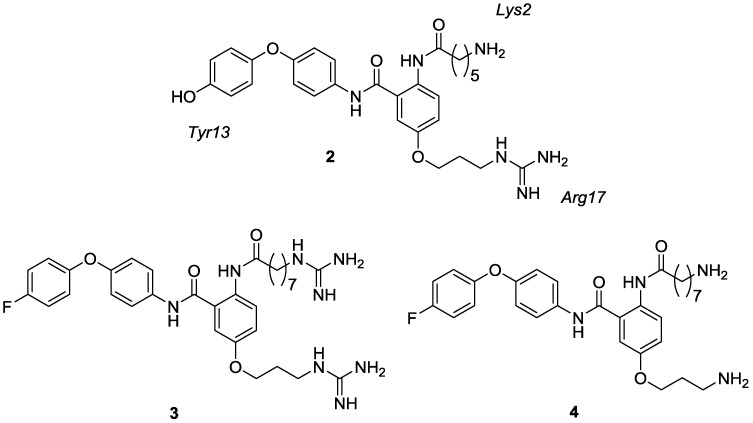
Structures of previously synthesised anthranilamide-based ω-conotoxin GVIA mimetics (**2**–**4**) [25,27].

As part of an ongoing program to develop new small-molecule inhibitors of Ca_v_2.2 channels, the pharmacophore of ω-conotoxin GVIA, a 27 residue peptide present in the venom of the cone snail *Conus geographus*, has been investigated. This peptide binds essentially irreversibly to the Ca_v_2.2 channel, making it unattractive as a therapeutic, however its well-defined structure has facilitated the development of peptidomimetics. A number of such mimetics have been disclosed [25,26,27,28,29], developed using the α,β-bond vector strategy described by Bartlett and Lauri [31], combined with interactive *de novo* design [32]. In these mimetics the biologically relevant tyrosine, lysine and arginine side chain mimics are projected from a central scaffold, as illustrated by the anthranilamide derivative (**2**) (Figure 2) [27,32]. Guided by the results of a radioligand-displacement assay, it has been concluded that with these anthranilamide-based mimetics (Compounds **2**–**4**, Figure 2) the optimum length of the alkyl side chains is *n =* 7 for the lysine mimic and *n =* 3 for the arginine mimic. It was also found that the replacement of the phenol functionality with fluorine is well tolerated [25]. The most potent fluorinated analogue in this series was found to be the diguanidino compound (**3**) while the corresponding diamino compound (**4**) had comparatively weak activity. This is consistent with previous results where truncation of the arginine side chain mimic to an amine in ω-conotoxin GVIA mimetics is typically detrimental to activity at the N-type channel [25,26,27,28,29].

**Figure 3:**
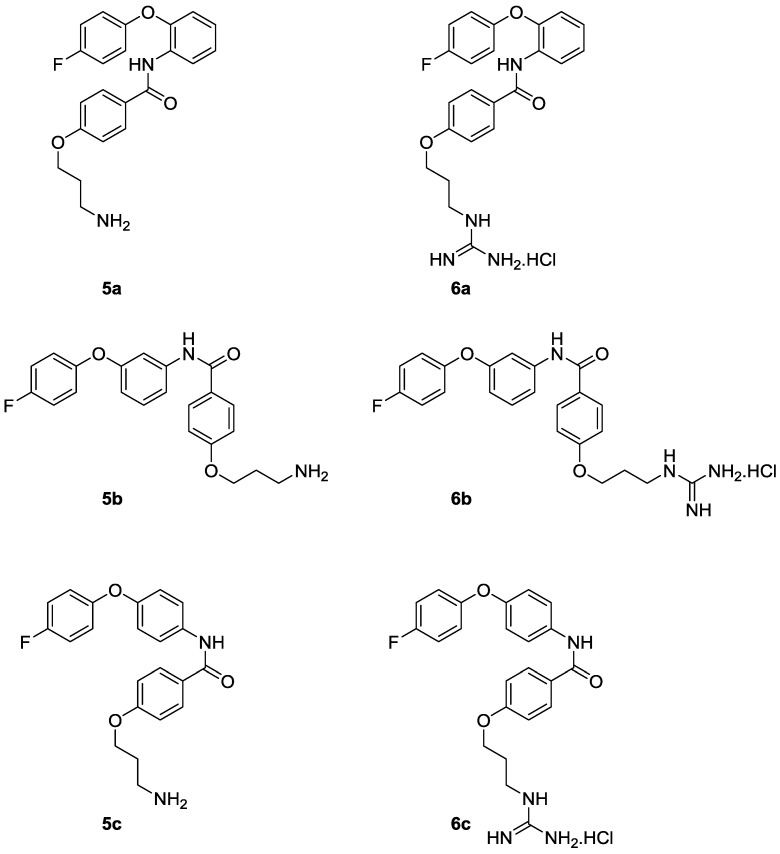
Analogues **5a**–**c** and **6a**–**c** targeted in this study.

In order to transition conotoxin mimics towards more drug-like compounds, a number of their physiochemical properties need to be adjusted. Marketed central nervous system (CNS) active drugs, for example, tend to have much lower molecular weights, percentage polar surface areas, total number of nitrogen and oxygen atoms, and hydrogen bond acceptors and donors than are found in mimetics like **2**. We have therefore embarked on a program of molecular modifications aimed at improving the physiochemical properties of this class of conotoxin mimics while retaining activity at the N-type calcium channel. A major priority has been to reduce overall molecular weight. Encouraged by favourable results obtained with the simplification of a benzothiazole class of mimetics, which involved the deletion of one of the amino acid side chain mimics [28], a similar strategy has been pursued with the anthranilamides. Thus, in the study described here, the effect on activity of the deletion of the lysine side chain mimic in compounds **2**–**4** has been investigated, together with the SAR related to the substitution pattern of the central aromatic ring (*ortho*, *meta* or *para*). The amino analogues and their corresponding monoguanidino analogues that were synthesised and tested in this study are shown in Figure 3, compounds **5a**–**c** and **6a**–**c**.

## 2. Results and Discussion

### 2.1. Chemistry

**Scheme 1:**
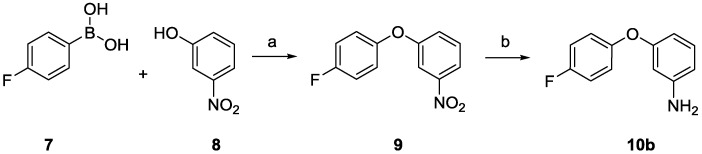
Synthesis of *meta*-(4-fluorophenoxy)aniline (**10b**). Reagents and conditions: (**a**) Cu(OAc)_2_, Et_3_N, air, 4 Å molecular sieves, dichloromethane (DCM), room temperature (RT), 24 h 83%; (**b**) Pd/C, NH_2_NH_2_·H_2_O, EtOH, RT, 4 h, 95%.

The previously described synthetic route to the anthranilamide-based mimics (**3** and **4**) [25] was modified to allow incorporation of the chloropropoxybenzamide moiety and its subsequent derivatisation. The *ortho* and *para* phenoxyl anilines (**10a** [33,34] and **10c** [25]) were readily available and the required *meta* phenoxyl aniline (**10b**) was synthesized in two steps from 4-fluorophenyl boronic acid (**7**) and *meta*-nitrophenol (**8**) via the intermediate phenoxynitrobenzene (**9**) (Scheme 1).

**Scheme 2:**
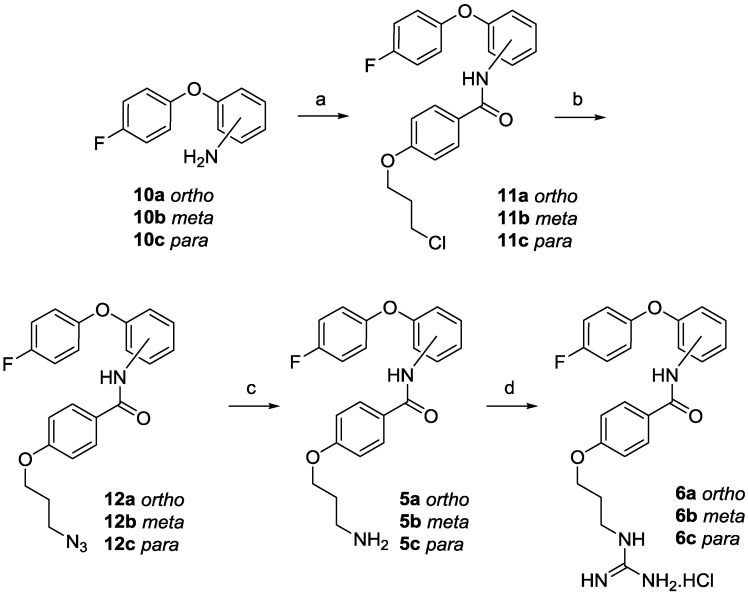
Synthesis of phenoxy anilides (**5a**–**c** and **6a**–**c**). Reagents and conditions: (**a**) 4-(3-chloropropoxy)benzoic acid, 1-(3-dimethylaminopropyl)-3-ethylcarbodiimide hydrochloride (EDC·HCl), 4-dimethylaminopyridine (DMAP), Et_3_N, DCM/tetrahydrofuran (THF), (**11a**) 47%, (**11b**) 81% or 4-(3-chloropropoxy)benzoyl chloride, THF, (**11c**) 70%; (**b**) NaN_3_, dimethyl sulfoxide (DMSO), 70 °C, (**12a**) 95%, (**12b**) 99%, (**12c**) 92%; (**c**) Pd/C, NH_2_NH_2_·H_2_O, MeOH, (**5a**–**c**) quant.; (**d**) 1*H*-pyrazole-1-carboximidine hydrochloride, *N*,*N*-diisopropylethylamine (DIPEA), dimethylformamide (DMF), (**6a**–**c**) quant.

With the required 4-fluorophenoxyanilines (**10a**–**c**) in hand, the desired *ortho*, *meta* and *para* amino phenoxy anilides (**5a**–**c**) and monoguanidino phenoxy anilides (**6a**–**c**) were synthesised, as outlined in Scheme 2. Reaction of the phenoxyl aniline (**10a**–**c**) with 4-(3-chloropropoxy)benzoic acid [35,36], using either carbodiimide activation [37] or formation of the acid chloride, gave the desired chloro compounds (**11a**–**c**). Subsequent conversion to the azide (**12a**–**c**) with sodium azide, followed by a transfer-hydrogenation reaction provided the corresponding amines (**5a**–**c**). Treatment of amines (**5a**–**c**) with 1*H*-pyrazole-carboxamidine [38] furnished the phenoxy anilides (**6a**–**c**) as hydrochloride salts.

### 2.2. Biology

It has been shown that SH-SY5Y neuroblastoma cells endogenously expressing human Ca_v_2.2 channels allow the rapid screening of potential channel blockers by means of a FLIPR assay [39]. The synthesised compounds, the amino (**5a**–**c**) and the guanidinium (**6a**–**c**) analogues, as well as the anthranilamide-based mimetic (**3**), were evaluated for their ability to inhibit Ca_v_2.2 calcium responses in SH-SY5Y cells in the presence of the L-type calcium channel blocker nifedipine. It was found that Ca^2+^ ion channel responses elicited by KCl-mediated depolarization were inhibited in a dose-dependent manner (Table 1). Compound **3** only partially inhibited responses at a concentration of 1 mM, resulting in an estimated IC_50_ value of 1452 µM. In contrast, **5a** and **5b** fully blocked KCl-induced Ca^2+^ responses with IC_50_s of 46 µM and 35 µM, respectively. In the guanidinium series, **6a** and **6b** retained weaker activity with IC_50_ values of 124 µM and 185 µM respectively. [40] Both *para* substituted compounds, **5c** and **6c**, were only weakly active and partially inhibited responses with IC_50_ values of 764 µM and 723 µM, respectively.

Compared to compound **3**, compounds **5a**–**c** and **6a**–**c** are significantly less complex and have molecular weights reduced by 33%–45%. It is encouraging, therefore, to find that all but **5c** and **6c** are considerably more active than **3**. A relationship between the substitution pattern around the central aromatic ring and biological activity can also be clearly seen, with the *ortho* and *meta* analogues showing considerably stronger activity than the *para* analogues. It is also interesting to note that the amino compounds **5a**–**b** are three to five fold more active than the guanidino compounds **6a**–**b**.

**Table 1:** Functional inhibition of calcium channels by compounds **3**, **5a**–**c**, **6a**–**c**.

Compound	logIC_50_	SEM	IC_50_ (µM)	95% CI (µM)
3 ^a^	~–2.84	0.29	1452 ^b^	380–5550
5a	–4.34	0.01	46	44–48
6a	–3.90	0.03	124 ^c^	107–150
5b	–4.45	0.01	35	33–37
6b	–3.73	0.02	185	169–203
5c	~–3.12	0.06	764 ^d^	575–1020
6c	–3.14	0.10	723 ^e^	447–1170

^a^ This compound gave an IC_50_ of 6 µM in a radioligand displacement assay with ^125^I-GVIA [25] but it has since been established that it is less potent in an assay of functional activity at Ca_v_2.2. [29]; ^b%^ Max inhibition, 15% at 300 µM; ^c%^ Max inhibition, 65% at 300 µM; ^d%^ Max inhibition, 30% at 300 µM; ^e%^ Max inhibition, 19% at 300 µM.

## 3. Experimental Section

### 3.1. Chemistry

#### 3.1.1. General Experimental Procedures

Starting materials and reagents were purchased from Sigma-Aldrich (Sydney, Australia) and used without purification. Solvents were dried, when necessary, using standard methods. Normal phase flash chromatography was performed on Merck silica gel No. 9385. Spectra were recorded on a Bruker Av400 or Av600 spectrometer (Fallanden, Switzerland). NMR spectra were referenced to residual solvent peak [chloroform (δ_H_ 7.26, δ_C_ 77.2), methanol (δ_H_ 4 .87, 3.30, δ_C_ 49.0)]. The units for all coupling constants (*J*) are in hertz (Hz). ^‡^Denotes signals only observed in 2D NMR. Mass spectrometry (APCI) was performed on a Thermo Scientific Q-Exactive FTMS. High-resolution mass spectra were recorded on a Waters Q-TOF II (Manchester, UK) or Thermo Scientific Q-Exactive FTMS mass spectrometer (Bremen, Germany). Melting points were recorded on a Stuart Scientific Melting Point Apparatus SMP3. Infrared spectra were recorded on a Perkin-Elmer RXI FTIR Spectrometer as thin films. Preparative HPLC was performed on a Waters Prep LC 4000 System using an Alltima C18 column (22 × 250 mm, 5 micron), detection at 237 nm. Mobile phase 12 mL/min 30% CAN/H_2_O/0.2% TFA isocratic for 135 min then 115 min gradient to 100% ACN containing 0.2% TFA.

#### 3.1.2. Synthesis

##### 4-(3-Chloropropoxy)-*N*-(2-(4-fluorophenoxy)phenyl)benzamide (**11a**)

Alkyl chloride **11a** was synthesised using a modified procedure outlined by Altin *et al.* [37]. A solution of 4-(3-chloropropoxy)benzoic acid [36] (1.27 g, 5.91 mmol) in dry THF (50 mL) was stirred under N_2_ at room temperature. Triethylamine (0.80 mL, 600 mg, 6.22 mmol) and DMAP (340 mg, 2.79 mmol) were added to the reaction mixture, followed by EDC·HCl (867 mg, 4.54 mmol). After 15 min a solution of the 2-(4-fluorophenoxy)aniline **10a** [33,34] (800 mg, 3.94 mmol) in dry DCM (20 mL) was added and the reaction mixture was stirred under N_2_ atmosphere at room temperature. After 48 h the THF was removed *in vacuo* before the residue was taken up in DCM (50 mL). The organic layer was washed with saturated NaHCO_3_ (3 × 50 mL), dried with Na_2_SO_4_ and concentrated to afford a brown oil. Purification by column chromatography (hexanes: EtOAc; 7:2) yielded the alkyl chloride **11a** as a colourless solid (711 mg, 47%). Mp: 101.5–103.5 °C. IR (ATR): 3333 s, 3113 w, 2942 w, 1653 s, 1600 s, 1497 s, 1446 s, 1310 m, 1250 s, 1196 s, 1173 s, 1036 m, 829 m, 764 m cm^−1^. ^1^H NMR (400 MHz, CDCl_3_): δ 8.59 (dd, *J* = 8.2, 1.5 Hz, 1H), 8.41 (br s, 1H), 7.81–7.77 (m, 2H), 7.19–7.14 (m, 1H), 7.09–7.01 (m, 5H), 6.97–6.94 (m, 2H), 6.83 (dd, *J* = 8.2, 1.5 Hz), 4.17 (t, *J* = 6.0 Hz, 2H), 3.75 (t, *J* = 6.0 Hz, 2H), 2.26 (quin, *J* = 6.0 Hz, 2H). ^13^C NMR (100 MHz, CDCl_3_): 165.0, 161.9, 159.4 (d, *J* = 241.3 Hz), 152.3, 146.4, 130.0, 129.2, 127.5, 124.3, 124.1, 121.1, 120.5 (d, *J* = 8.3 Hz), 117.3, 116.8 (d, *J* = 23.5 Hz), 114.7, 64.7, 41.4, 32.2. ^19^F NMR (376 MHz, CDCl_3_): δ −119.4. LRMS (APCI): *m/z* 400.1 [M + H]^+^ (100%), 288.1 [M − C_6_H_4_FO]^+^ (48%). HRMS (APCI): *m/z* calcd. For C_22_H_19_^35^ClFNO_3_ [M + H]^+•^: 399.1032, found: 399.1032.

##### 4-(3-Azidopropoxy)-*N*-(2-(4-fluorophenoxy)phenyl)benzamide (**12a**)

The azide **12a** was synthesised according to a procedure outlined by Alvarez *et al.* [41] Sodium azide (95 mg, 1.5 mmol) was added to a solution of alkyl chloride **11a** (0.45 g, 1.1 mmol) in DMSO (3 mL) and stirred under N_2_ atmosphere at 70 °C. After 48 h, the reaction mixture was allowed to cool and DCM (30 mL) was added. (CAUTION: It is recommended that DCM be substituted with diethyl ether for larger scale reactions, to avoid the formation of hazardous side products such as azido-chloromethane and diazidomethane) The organic layer was washed with brine (5 × 50 mL), dried with Na_2_SO_4_ and concentrated to provide azide **12a** as a pale brown solid (377 mg, 95%). ^1^H NMR spectroscopy deemed this solid to be pure enough to use in the next step. Mp: 69.2–71.2 °C. IR (ATR): 3430 s, 3071 w, 2935 w, 2093 s, 1668 s, 1602 s, 1499 s, 1442 s, 1309 m, 1244 s, 1194 s, 1036 w, 841 m, 758 m cm^−1^. ^1^H NMR (400 MHz, CDCl_3_): δ 8.59 (dd, *J* = 8.2, 1.6 Hz, 1H), 8.41 (br s, 1H), 7.81–7.77 (m, 2H), 7.18–7.14 (m, 1H), 7.09–7.00 (m, 5H), 6.96–6.93 (m, 2H), 6.83 (dd, *J* = 8.2, 1.4 Hz, 1H), 4.10 (t, *J* = 6.0 Hz, 2H), 3.52 (t, *J* = 6.0 Hz, 2H), 2.07 (quin, *J* = 6.0 Hz, 2H). ^13^C NMR (100 MHz, CDCl_3_): δ 164.9, 161.7, 159.3 (d, *J* = 241.4 Hz), 152.3, 146.4, 129.9, 129.1, 127.5, 124.3, 124.1, 121.0, 120.4 (d, *J* = 8.3 Hz), 117.2, 116.7 (d, *J* = 23.3 Hz), 114.6, 64.9, 48.2, 28.8. ^19^F NMR (376 MHz, CDCl_3_): δ −119.4. LRMS (APCI): *m/z* 407.2 [M + H]^+^ (100%), 204.1 [M − C_10_H_8_N_3_O_2_]^+^ (39%), 381.2 [M − N_2_ + 3H]^+^ (26%). HRMS (APCI): *m/z* calcd. For C_22_H_19_FN_4_O_3_ [M]^+•^: 406.1436, found: 406.1438.

##### 4-(3-Aminopropoxy)-*N*-(2-(4-fluorophenoxy)phenyl)benzamide (**5a**)

A solution of azide **12a** (100 mg, 0.25 mmol), 10% Pd/C (12 mg) and hydrazine monohydrate (30 µL, 30 mg, 0.62 mmol) in MeOH (3 mL) was vigorously stirred under N_2_ at room temperature. After 20 min, the reaction mixture was filtered through Celite™ and concentrated to provide the amino compound **5a** as yellow oil in a quantitative crude yield. A small amount was purified by reversed-phase HPLC to give a sample for biological testing. IR (ATR): 3432 br, 3071 w, 2942 w, 2873 w, 1653 s, 1601 s, 1493 s, 1378 m, 1248 s, 1203 s, 1173 s, 838 m, 758 m cm^−1^. ^1^H NMR (600 MHz, CD_3_OD): δ 7.90–7.89 (m, 1H), 7.76–7.74 (m, 2H), 7.18–7.17 (m, 2H), 7.05–6.99 (m, 7H), 4.08 (t, *J* = 6.2 Hz, 2H), 2.81 (t, *J* = 7.0 Hz, 2H), 1.95–1.91 (m, 2H). ^13^C NMR (150 MHz, CD_3_OD): δ 168.2, 163.5, 160.7 (d, *J* = 238.9 Hz), 154.3, 150.8, 130.5, 130.4, 127.6, 127.4, 126.7, 125.0, 120.8 (d, *J* = 7.8 Hz), 120.0, 117.2 (d, *J* = 23.6 Hz), 115.3, 67.3, 39.6, 33.3. ^19^F NMR (376 MHz, CD_3_OD): δ −120.5. LRMS (APCI): *m/z* 381.2 [M + H]^+^ (50%), 380.2 [M]^+^ (100%). HRMS (APCI): *m/z* calcd. For C_22_H_21_FN_2_O_3_ [M]^+•^: 380.1531, found: 380.1532.

##### *N*-(2-(4-Fluorophenoxy)phenyl)-4-(3-guanidinopropoxy)benzamide hydrochloride (**6a**)

The guanidinylated compound **6a** was synthesized according to a modified procedure by Bernatowicz *et al.* [38]. Amine **5a** (102 mg, 0.24 mmol), DIPEA (42 µL, 31 mg, 0.24 mmol), 1H-pyrazole-1-carboximidine hydrochloride (35 mg, 0.24 mmol) and DMF (2 mL) were combined and stirred vigorously under N_2_ atmosphere at room temperature. After 18 h TLC analysis revealed that starting material remained, additional 1H-pyrazole-1-carboximidine hydrochloride (9 mg, 0.06 mmol) and DIPEA (10 µL, 8 mg, 0.06 mmol) were added. After a further 24 h the solvent was removed in vacuo to yield the guanidinylated compound **6a** as crystalline product. This solid was dissolved in MeOH (1 mL) and the product precipitated by addition of Et_2_O (10 mL). After removal of the residual solvent the product was obtained as a crystalline solid (47 mg, 39%) IR (ATR): 3307 br s, 3251 br s, 3139 br s, 1643 s, 1601 s, 1497 s, 1443 s, 1245 s, 1196 s, 1174 s, 840 m, 759 m cm^−1^. ^1^H NMR (400 MHz, CD_3_OD): δ 7.88–7.85 (m, 1H), 7.78–7.76 (m, 2H), 7.21–7.18 (m, 2H), 7.07–6.98 (m, 6H), 6.97–6.94 (m, 1H), 4.13 (t, *J* = 5.9 Hz, 2H), 3.41 (t, *J* = 6.8 Hz, 2H), 2.11-2.05 (m, 2H). ^13^C NMR (100 MHz, CD_3_OD): δ 168.2, 163.2, 160.2 (d, *J* = 239 Hz), 158.7, 154.4, 151.0, 130.5, 130.4, 127.9, 127.6, 126.9, 125.0, 120.8 (d, *J* = 8.3 Hz), 120.1, 117.2 (d, *J* = 23.4 Hz), 115.4, 66.3, 39.5, 29.5. ^19^F NMR (376 MHz, CD_3_OD): δ −120.6. LRMS (APCI): *m/z* 422.2 [M]^+^ (62%), 380.15 [M − CH_2_N_2_]^+^ (100%). HRMS (ESI): *m/z* calcd. For C_23_H_23_FN_4_O_3_ [M]^+•^: 422.1749, found: 422.1750.

##### 1-(4-Fluorophenoxy)-3-nitrobenzene (**9**)

The fluoro diaryl ether **9** was synthesised according to a general procedure outlined by Evans *et al.* [42] 4-Fluorophenyl boronic acid **7** (300 mg, 2.16 mmol), *m*-nitrophenol **8** (200 mg, 1.44 mmol), Cu(OAc)_2_ (260 mg, 1.44 mmol) and freshly activated powdered 4 Å molecular sieves were added to dry DCM (15 mL). Et_3_N (1.0 mL, 730 mg, 7.19 mmol) was then added to the reaction mixture. The reaction left open to air through a drying tube and stirred at room temperature, and reaction progress was monitored by TLC (hexanes: EtOAc; 30:1). After 24 h, the resulting slurry was filtered through Celite™ and concentrated to give a brown oil. The crude product was purified by flash column chromatography (hexanes: EtOAc; 30:1) to give the fluoro diaryl ether **9** as a yellow oil (280 mg, 83%). IR (ATR): 1526 s, 1497 s, 1473 s, 1352 s, 1212 s, 1187 s, 811 s, 734 s cm^−1^; ^1^H NMR (400 MHz, CDCl_3_): δ 7.93 (ddd, *J* = 8.2, 2.3, 0.9 Hz, 1H), 7.75 (t, *J* = 2.3 Hz, 1H), 7.48 (t, *J* = 8.2 Hz, 1H) 7.29 (ddd, *J* = 8.2, 2.3, 0.9 Hz, 1H), 7.13–7.08 (m, 2H), 7.07–7.02 (m, 2H). ^13^C NMR (100 MHz, CDCl_3_) 159.8 (d, *J* = 242.4 Hz), 159.0, 151.3, 149.5, 130.5, 123.8, 121.7 (d, *J* = 8.4 Hz), 117.8, 117.1 (d, *J* = 23.2 Hz), 112.4. ^19^F NMR (376 MHz, CDCl_3_): δ −118.1. LRMS (APCI): *m/z* 234.1 [M + H]^+^ (100%), 204.1 [M − O_2_ + 2H]^+^ (32%), 188.1 [M − NO_2_]^+^ (24%). HRMS (APCI): *m/z* calcd. for C_12_H_10_FNO_3_ [M + H]^+^: 234.0561, found: 234.0562.

##### 3-(4-Fluorophenoxy)aniline (**10b**)

The *m*-aniline **10b** was prepared following a procedure similar to that of Bavin *et al.* [43] Hydrazine monohydrate (790 mg, 0.77 mL, 16 mmol) was added to a deoxygenated mixture of the nitroarene **9** (0.50 g, 2.1 mmol), 10% Pd/C (50 mg) and EtOH (25 mL). This reaction mixture was then refluxed under N_2_ until TLC analysis (hexanes:EtOAc; 4:1) showed no starting material (typically 4 h). The reaction mixture was allowed to cool, filtered through Celite™, concentrated *in vacuo* and the colourless residue purified by column chromatography (hexanes: EtOAc; 4:1) to yield the *m*-aniline **10b** as a colourless oil (410 mg, 95%). All spectral data was in accordance with that in the literature [44]. IR (ATR): 3462 s, 3317 s, 1619 m, 1584 m, 1485 s, 1282 m, 1193 s, 1141 s, 832 m, 772 m cm^−1^. ^1^H NMR (400 MHz, CDCl_3_): δ 7.08 (t, *J* = 8.0, 1H) 7.04–6.96 (m, 4H) 6.42 (ddd, *J* = 8.0, 2.2, 0.8 Hz, 1H), 6.35 (ddd, *J* = 8.0, 2.2, 0.8, 1H), 6.30 (t, *J* = 2.2 Hz, 1H), 3.70 (bs, 2H). ^13^C NMR (100 MHz, CDCl_3_): δ 159.0, 158.7 (d, *J* = 239.8 Hz), 152.9, 148.2, 130.5, 120.8 (d, *J* = 8.3 Hz), 116.3 (d, *J* = 23.1 Hz), 110.1, 108.3, 105.0. ^19^F NMR (376 MHz, CDCl_3_): δ −120.5. LRMS (APCI): *m/z* 204.1 [M + H]^+^ (100%). HRMS (APCI): *m/z* calcd. for C_12_H_10_FNO [M + H]^+^: 204.0819, found: 204.0820.

##### 4-(3-Chloropropoxy)-*N*-(3-(4-fluorophenoxy)phenyl)benzamide (**11b**)

The alkyl chloride **11b** was synthesised using a modified procedure outlined by Altin *et al.* [37]. A solution of 4-(3-chloropropoxy)benzoic acid [33,34] (910 mg, 4.24 mmol), in dry THF (50 mL) was stirred under N_2_ at at room temperature. Et_3_N (0.87 mL, 630 mg, 6.22 mmol), and DMAP (340 mg, 2.79 mmol) were then added to the reaction mixture, followed by EDC·HCl (867 mg, 4.53 mmol). After 15 min, a solution of the 3-(4-fluorophenoxy)aniline **10b** (575 mg, 2.83 mmol) in dry DCM (20 mL) was added, and the reaction mixture was stirred under a N_2_ atmosphere at room temperature. After 48 hours the THF was removed *in vacuo* before being taken up into DCM (50 mL). The organic layer was then washed with NaHCO_3_ (3 × 50 mL), dried with Na_2_SO_4_ and concentrated to afford a light brown oil. Purification by column chromatography (hexanes: EtOAc; 7:2) yielded the alkyl chloride **11b** as a colourless solid (917 mg, 81%). Mp: 116.5–117.0 °C. IR (ATR): 3366 s, 1652 s, 1601 s, 1528 m, 1500 s, 1442 s, 1267 m, 1246 s, 1196 s, 846 m, 773 s cm^−1^. ^1^H NMR (400 MHz, CDCl_3_): δ 7.82–7.89 (m, 2H), 7.75 (bs, 1H), 7.35–7.27 (m, 3H), 7.06–7.00 (m, 4H), 6.98–6.94 (m, 2H), 6.74 (dt, *J* = 7.2, 2.0 Hz, 1H), 4.18 (t, *J* = 6.0 Hz, 2H), 3.76 (t, *J* = 6.0 Hz, 2H), 2.27 (quin, *J* = 6.0 Hz, 2H). ^13^C NMR (100 MHz, CDCl_3_): δ 165.2, 161.9, 159.1 (d, *J* = 240.4 Hz), 158.5, 152.7, 139.7, 130.3, 129.1, 127.3, 121.0 (d, *J* = 8.3 Hz), 116.5 (d, *J* = 23.0 Hz), 114.8, 144.7, 114.1, 110.2, 64.7, 41.4, 32.2. ^19^F NMR (376 MHz, CDCl_3_): δ −120.2. LRMS (APCI): *m/z* 400.1 [M + H]^+^ (100%). HRMS (APCI): *m/z* calcd. for C_22_H_20_ClFNO_3_ [M + H]^+^: 400.1110, found: 400.1113.

##### 4-(3-Azidopropoxy)-*N*-(3-(4-fluorophenoxy)phenyl)benzamide (**12b**)

The azide **12b** was synthesised according to a procedure outlined by Alvarez *et al.* [41]. Sodium azide (219 mg, 3.38 mmol) was added to a solution of the alkyl chloride **11b** (900 mg, 2.25 mmol) in DMSO (4 mL) and stirred under N_2_ at 70 °C. After 48 h, the reaction was allowed to cool and DCM (40 mL) was added. (CAUTION: It is recommended that DCM be substituted with diethyl ether for larger scale reactions, to avoid the formation of hazardous side products, such as azido-chloromethane and diazidomethane) The organic layer was washed with brine (5 × 100 mL), dried with Na_2_SO_4_ and concentrated to provide the azide **12b** as a colourless solid (902 mg, 99%), which was deemed sufficiently pure by ^1^H NMR spectroscopy for use in the next step. Mp: 104.6–106.2 °C. IR (ATR): 3366 s, 2104 s, 1640 s, 1593 s, 1499 s, 1441 s, 1248 s, 1199 s, 848 m, 770 m cm^−1^. ^1^H NMR (400 MHz, CDCl_3_): δ 7.82–7.79 (m, 2H), 7.76 (bs, 1H), 7.35–7.27 (m, 3H), 7.06–7.00 (m, 4H), 6.97–6.93 (m, 2H), 6.74 (dt, *J* = 7.0, 2.2 Hz, 1H), 4.11 (t, *J* = 6.3 Hz, 2H), 3.53 (t, *J* = 6.3 Hz, 2H), 2.08 (quin, *J* = 6.3 Hz, 2H). ^13^C NMR (100 MHz, CDCl_3_): δ 165.3, 161.9, 159.1 (d, *J* = 240.5 Hz), 158.6, 152.7, 139.7, 130.3, 129.1, 127.3, 121.0 (d, *J* = 8.4 Hz), 116.5 (d, *J* = 23.2 Hz), 114.8, 114.7, 114.2, 110.2, 65.0, 48.3, 28.9. ^19^F NMR (376 MHz, CDCl_3_): δ −120.2. LRMS (APCI): *m/z* 407.2 [M + H]^+^ (100%). HRMS (APCI): *m/z* calcd. For C_22_H_20_FN_4_O_3_ [M + H]^+^: 407.1514, found: 407.1514.

##### 4-(3-Aminopropoxy)-*N*-(3-(4-fluorophenoxy)phenyl)benzamide (**5b**)

A solution of the azide **12b** (100 mg, 0.25 mmol), 10% Pd/C (12 mg) and hydrazine monohydrate (30 µL, 30 mg, 0.62 mmol) in MeOH (3 mL) were vigorously stirred under a N_2_ atmosphere at room temperature. After 20 mins, the reaction mixture was filtered through Celite™ and concentrated to provide the amino compound **5b** as a colourless oil in a quantitative crude conversion. IR (ATR): 3280 br s, 1647 s, 1597 m, 1540 m), 1484 s, 1244 s, 1195 s, 837 s, 779 s, 760 s, 684 s cm^−1^.^1^H NMR (400 MHz, CD_3_OD): δ 7.90–7.87 (m, 2H), 7.45 (t, *J* = 2.0 Hz, 1H), 7.39 (ddd, *J* = 8.0, 2.1, 1.0 Hz, 1H), 7.31 (t, *J* = 8.0 Hz, 1H), 7.13–7.01 (m, 6H), 6.73 (ddd, *J* = 8.0, 2.1, 1.0 Hz, 1H), 4.13 (t, *J* = 6.6 Hz, 2H), 2.84 (br t, *J* = 6.6 Hz, 2H), 1.96 (quin, 6.6 Hz, 2H). ^13^C NMR (100 MHz, CD_3_OD): δ 168.4, 163.5, 160.3 (d, *J* = 238.9 Hz), 159.5, 154.3, 141.6, 130.9, 130.6, 128.0, 121.9 (d, *J* = 8.3 Hz), 117.3 (d, *J* = 23.5 Hz), 116.7, 115.3, 114.9, 112.0, 67.3, 39.6, 33.0. ^19^F NMR (376 MHz, CD_3_OD): δ −120.5. LRMS (APCI): *m/z* 380.2 [M]^+^ (100%), 381.2 [M + H]^+^ (41%). HRMS (APCI): *m/z* calcd. For C_22_H_21_FN_2_O_3_ [M]^+^: 380.1532, found: 380.1531.

##### *N*-(3-(4-Fluorophenoxy)phenyl)-4-(3-guanidinopropoxy)benzamide hydrochloride (**6b**)

The guanidinylated compound **6b** was synthesised according to a modified procedure by Bernatowicz *et al.* [38]. The amine **5b** (47 mg, 0.12 mmol), DIPEA (35 µL, 26 mg, 0.20 mmol), 1*H*-pyrazole-1-carboximidine hydrochloride **27** (25 mg, 0.17 mmol) and DMF (2 mL) were added to a flask and stirred vigorously under a nitrogen atmosphere at room temperature. After 18 h, the solvent was removed *in vacuo* to yield the guanidinylated compound **6b** as a colourless solid in quantitative conversion. IR (ATR): 3244 br s, 3150 br s, 1651 s, 1592 s, 1503 s, 1479 s, 1249 s, 1210 m, 1171 s, 826 m, 791 m, 692 m cm^−1^. ^1^H NMR (400 MHz, CD_3_OD): δ 7.92–7.88 (m, 2H), 7.47 (t, *J* = 2.2 Hz, 1H), 7.41 (ddd, *J* = 8.1, 2.2, 0.9 Hz, 1H), 7.30 (t, *J* = 8.1 Hz, 1H), 7.11–7.01 (m, 6H), 6.72 (ddd, *J* = 8.1, 2.2, 0.9 Hz, 1H), 4.16 (t, *J* = 6.3 Hz, 2H), 3.42 (t, *J* = 6.3 Hz, 2H), 2.09 (quin, *J* = 6.3 Hz, 2H). ^13^C NMR (100 MHz, CD_3_OD): 168.3, 163.2, 160.3 (d, *J* = 239.0 Hz), 159.9, 158.8, 154.3, 141.6, 130.9, 130.7, 128.4, 121.9 (d, *J* = 8.4 Hz), 117.3 (d, *J* = 23.3 Hz), 116.7, 115.3, 115.0, 112.0, 66.3, 39.6, 29.6. ^19^F NMR (376 MHz, CD_3_OD): δ −120.5. LRMS (APCI): *m/z* 422.2 [M]^+^ (32%), 381.2 [M − CHN_2_]^+^ (35%), 380.2 [M − CH_2_N_2_]^+^ (100%). HRMS (APCI): *m/z* calcd. For C_23_H_23_FN_4_O_3_ [M]^+^: 422.1749, found: 422.1749.

##### 4-(3-Chloropropoxy)-*N*-(4-(4-fluorophenoxy)phenyl)benzamide (**11c**)

4-(3-Chloropropoxy)benzoic acid (790 mg, 3.68 mmol) was taken up into SOCl_2_ (2.00 mL, 3.28 g, 27.5 mmol) and the reaction mixture was stirred for 30 min. Excess SOCl_2_ was removed under a stream of N_2_ to provide an acid chloride intermediate. A solution of 4-(4-fluorophenoxy)aniline **10c** (650 mg, 3.20 mmol) in dry THF (35 mL) was added, and the reaction mixture was stirred at room temperature for 18 h. The THF was removed *in vacuo*, and the resultant solid was taken up into EtOAc (60 mL) and washed with sat. NaHCO_3_ (3 × 100 mL). The organic layer was dried with Na_2_SO_4_ and concentrated to afford the crude alkyl chloride **11c** as a brown solid. The solid was recrystallised from hot EtOAc to give the desired alkyl chloride **11c** as a colourless solid (900 mg, 70%). Mp: 147.3–148.1 °C. IR (ATR): 3338 s, 1642 s, 1607 m, 1496 s, 1259 s, 852 s, 833 s, 816 s, 761 s cm^−1^. ^1^H NMR (400 MHz, CDCl_3_): δ 7.88–7.84 (m, 2H), 7.71 (br s, 1H), 7.61–7.58 (m, 2H), 7.07–6.97 (m, 8H), 4.22 (t, *J* = 6.0 Hz, 2H), 3.79 (t, *J* = 6.0 Hz, 2H), 2.30 (quin, *J* = 6.0 Hz, 2H). ^13^C NMR (100 MHz, CDCl_3_): δ 165.3, 161.9, 158.9^‡^ (d, *J* = 239.7 Hz), 154.2, 153.4^‡^, 133.7, 129.1, 127.4, 122.2, 120.2 (d, *J* = 8.2 Hz), 119.3, 116.4 (d, *J* = 23.1 Hz), 114.7, 64.7, 41.5, 32.2. ^19^F NMR (376 MHz, CDCl_3_): δ −120.8. LRMS (APCI): *m/z* 400.1 [M + H]^+^ (75%), 399.1 [M]^+^ (100%). HRMS (APCI): *m/z* calcd. for C_22_H_19_ClFNO_3_ [M]^+^: 399.1032, found: 399.1032.

##### 4-(3-Azidopropoxy)-*N*-(4-(4-fluorophenoxy)phenyl)benzamide (**12c**)

The azide **12c** was synthesised according to a procedure outlined by Alvarez *et al.* [41]. Sodium azide (63 mg, 0.97 mmol) was added to a solution of the alkyl chloride **11c** (300 mg, 0.752 mmol) in DMSO (4 mL) and stirred under N_2_ at 70 °C. After 48 h, the reaction mixture was cooled and DCM (40 mL) was added. (CAUTION: It is recommended that DCM be substituted with diethyl ether for larger scale reactions, to avoid the formation of hazardous side products, such as azido-chloromethane and diazidomethane) The organic layer was washed with brine (5 × 100 mL), dried with Na_2_SO_4_ and concentrated to provide the azide **12c** as a colourless solid (279 mg, 92%), which was deemed sufficiently pure by ^1^H NMR spectroscopy for use in the next step. Mp: 120.7–122.0 °C. IR (ATR): 3325 s, 2099 s, 1648 s, 1605 m, 1494 s, 1250 s, 1208 s, 828 s, 764 m cm^−1^. ^1^H NMR (400 MHz, CDCl_3_): δ 7.86–7.82 (m, 2H), 7.68 (br s, 1H), 7.60–7.56 (m, 2H), 7.05–6.95 (m, 8H), 4.13 (t, *J* = 6.0 Hz, 2H), 3.54 (t, *J* = 6.0 Hz, 2H), 2.09 (quin, *J* = 6.0 Hz, 2H). ^13^C NMR (100 MHz, CDCl_3_): δ 165.3, 161.8, 158.8^‡^ (d, *J* = 261.0 Hz), 154.3, 153.4^‡^, 133.7, 129.1, 127.4, 122.2, 120.2 (d, *J* = 8.3 Hz), 119.3, 116.4 (d, *J* = 23.2 Hz), 114.7, 65.0, 48.3, 28.9. ^19^F NMR (376 MHz, CDCl_3_): δ −120.8. LRMS (APCI): *m/z* 406.1 [M]^+^ (100%). HRMS (APCI): *m/z* calcd. for C_22_H_19_FN_4_O_3_ [M]^+^: 406.1436, found: 406.1438.

##### 4-(3-Aminopropoxy)-*N*-(4-(4-fluorophenoxy)phenyl)benzamide (**5c**)

A solution of the azide **11c** (100 mg, 0.25 mmol), 10% Pd/C (12 mg) and hydrazine monohydrate (30 µL, 30 mg, 0.62 mmol) in MeOH (3 mL) were vigorously stirred under a N_2_ atmosphere at room temperature. After 20 mins, the reaction mixture was filtered through Celite™ and concentrated to provide the amino compound **5c** as a yellow oil in a quantitative crude conversion. IR (ATR): 3307 m, 3282 m, 1632 s, 1606 m, 1494 s, 1248 m, 1208 s, 820 s, 758 s cm^−1^. ^1^H NMR (400 MHz, CD_3_OD): δ 7.92–7.89 (m, 2H), 7.66–7.62 (m, 2H), 7.10–6.95 (m, 8H), 4.14 (t, *J* = 6.2 Hz, 2H), 2.84 (t, *J* = 7.0 Hz, 2H), 2.00–1.93 (m, 2H). ^13^C NMR (100 MHz, CD_3_OD): δ 168.3, 163.5, 160.1 (d, *J* = 238.7 Hz), 155.5, 154.9, 135.5, 130.5, 128.1, 124.2, 121.2 (d, *J* = 8.1 Hz), 119.8, 117.2 (d, *J* = 23.3 Hz), 115.3, 67.3, 39.6, 33.3. ^19^F NMR (376 MHz, CD_3_OD): δ −121.0. LRMS (APCI): *m/z* 381.2 [M + H]^+^ (28%), 380.2 [M + H]^+^ (100%), 323.1 [M − C_3_H_7_N]^+^ (34%). HRMS (APCI): *m/z* calcd. for C_22_H_21_FN_2_O_3_ [M]^+^: 380.1531, found: 380.1533.

##### *N*-(4-(4-Fluorophenoxy)phenyl)-4-(3-guanidinopropoxy)benzamide hydrochloride (**6c**)

The guanidinylated compound **6c** was synthesised according to a modified procedure by Bernatowicz *et al.* [38]. The amine **5c** (47 mg, 0.12 mmol), DIPEA (35 µL, 26 mg, 0.20 mmol), 1*H*-pyrazole-1-carboximidine hydrochloride (25 mg, 0.17 mmol) and DMF (2 mL) were added to a flask and stirred vigorously under a nitrogen atmosphere at room temperature. After 18 h, the solvent was removed *in vacuo* to yield the guanidinylated compound **6c** as a yellow oil with quantitative conversion. IR (ATR): 3272 m, 3185 m, 1631 s, 1601 s, 1495 s, 1437 s, 1393 s, 1247 s, 1208 s, 1177 s, 839 m, 759 m. ^1^H NMR (600 MHz, CD_3_OD): δ 7.94–7.92 (m, 2H), 7.65–7.63 (m, 2H), 7.10–6.99 (m, 6H), 6.98–6.95 (m, 2H), 4.16 (t, *J* = 5.9 Hz, 2H), 3.43 (t, *J* = 6.8 Hz, 2H), 2.13–2.07 (m, 2H). ^13^C NMR (150 MHz, CD_3_OD): δ 168.2, 163.1, 159.5 (d, *J* = 240.9 Hz), 158.7, 155.5, 154.9, 135.4, 130.6, 128.3, 124.2, 121.2 (d, *J* = 8.0 Hz), 119.8, 117.2 (d, *J* = 24.1 Hz), 115.3, 66.3, 39.5, 29.6. ^19^F NMR (376 MHz, CD_3_OD): δ −120.9. LRMS (APCI): *m/z* 422.2 [M]^+^ (14%), 381.2 [M − CN_2_H] ^+^ (30%), 380.2 [M − CH_2_N_2_]^+^ (100%), 323.1 [M-C_3_H_9_N_3_]^+^ (31%). HRMS (APCI): *m/z* calcd. for C_23_H_23_FN_4_O_3_ [M]^+^: 422.1749, found: 422.1750.

### 3.2. Biology

#### Fluorescence Measurement of Calcium Responses

SH-SY5Y cells were plated at a density of 30,000–50,000 cells/well on 384-well black-walled imaging plates and loaded for 30 min at 37 °C with Calcium 4 no-wash dye (Molecular Devices, Sunnyvale, CA, USA) diluted in physiological salt solution (PSS; composition: 140 mM NaCl, 11.5 mM glucose, 5.9 mM KCl, 1.4 mM MgCl_2_, 1.2 mM NaH_2_PO_4_, 5 mM NaHCO_3_, 1.8 mM CaCl_2_, 10 mM 4-(2-hydroxyethyl)-1-piperazineethanesulfonic acid (HEPES), pH 7.4). Calcium responses, elicited by addition of 90 mM KCl and 5 mM CaCl_2_ in the presence of 10 µM nifedipine, were measured using a FLIPR^TETRA^ fluorescent plate reader (excitation, 470–495 nm; emission, 515–575 nm) after 5 min pre-treatment with test compounds. Fluorescent responses were plotted as response over baseline using ScreenWorks (Molecular Devices, version 3.1.1.4). Concentration-response curves of Calcium responses, normalized to control responses, were generated using GraphPad Prism (Version 4.00, San Diego, CA, USA) using a 4-parameter Hill equation with variable Hill slope and bottom >0 fitted to the data.

## 4. Conclusions

Despite being significantly less complex than the originally designed anthranilamide ω-conotoxin GVIA mimetics (e.g., **2**), the simplified fluorophenoxyanilides described here (**5a**–**c** and **6a**–**c**) show enhanced activity in the SH-SY5Y FLIPR assay. The compounds with *para*-substitution around the central ring (**5c** and **6c**) were found to have the weakest activity, suggesting that some type of pre-organisation through restricted rotation might enhance the activity of the *ortho* and *meta* analogues (**5a**, **5b**, **6a** and **6b**). It is also unusual for the amines to be more active than the guanidines in this class of channel blocker. While primary amines typically do not make good drugs, this observation does open the possibility of developing N-type channel blockers capable of crossing the blood brain barrier. Compounds bearing very strongly basic functional groups like guanidine are unlikely to cross the blood brain barrier [45] whereas there are many CNS-active drugs that bear tertiary amines. The mode of action of the compounds reported here is currently being investigated in patch clamp electrophysiology experiments, the results of which will be reported in due course.
